# The Predictors of High Dietary Salt Intake among Hypertensive Patients in Iran

**DOI:** 10.1155/2020/6748696

**Published:** 2020-04-07

**Authors:** Parvin Reyhani, Fariba Azabdaftari, Mehrangiz Ebrahimi-Mamagani, Mohammad Asghari-Jafarabadi, Behjat Shokrvash

**Affiliations:** ^1^Department of Health Education and Promotion, Faculty of Health, Tabriz University of Medical Sciences, Tabriz, Iran; ^2^Basic Sciences Department, Paramedical School, Tabriz University of Medical Sciences, University Campus, Danshgah Street, Tabriz, Iran; ^3^Social Determinant of Health Research Center, Tabriz University of Medical Sciences, Tabriz, Iran; ^4^Department of Biochemistry and Diet Therapy, School of Nutrition and Food Sciences, Tabriz University of Medical Sciences, Tabriz, Iran; ^5^Road Trafic Injury Research Center, Tabriz University of Medical Sciences, Tabriz, Iran; ^6^Department of Statistics and Epidemiology, Faculty of Health, Tabriz University of Medical Sciences, Tabriz, Iran; ^7^Medical Education Research Center, Health Management and Safety Promotion Research Institute, Tabriz University of Medical Sciences, Tabriz, Iran

## Abstract

**Background:**

Hypertension and its complications are among the major health problems all over the world, particularly in developing countries. The aims of this study were to show that, weather the hypertensive patients follow the expertise recommendations and differences between men and women in their levels of knowledge and behavior in salt taking.

**Methods:**

The present cross-sectional study was conducted among all hypertensive patients in rural health centers of Tabriz, Iran, in Feb–May; 2016. Data were collected by an interviewer-administrated questionnaire, along with anthropometric, blood pressure, and 24-hour urinary sodium excretion measurements. Multivariate logistic regression analysis was used to compute adjusted odds ratio (OR).

**Results:**

In all 205 patients, 62.9% female, 40.5% aged over 60 years, and 53.7% with low or moderate socioeconomic status, 49.3% body mass index (BMI) 30 kg/m^2^ and above, 10.2% of the patients had systolic/diastolic equal and above (≥) 40/90 mmHg, participated in the study. In total, 16.6% were aware of the daily salt allowance for healthy people with sex difference (*P* < 0.001). Significant predictors of adding salt beyond the dietary recommendations in food preparation were occupation (unemployed) (OR = 4.05, 95% CI = 1.041–15.78, (*P* = 0.044)) and blood pressure level (systolic/diastolic ≥140/90) (OR = 2.76, 95% CI = 1.28–5.96 (*P* = 0.010)), while adding salt at the table correlated with sex (men) (OR = 4.47, 95% CI = 1.21–16.57 (*P* = 0.025)), age (54–59) (OR = 0.05 95% CI = 0.01–0.39, (*P* = 0.005)), and knowledge (general) (OR = 1.06, 95% CI = 0.99–1.13 (*P* = 0.05)).

**Conclusion:**

The different pattern of salt intake was observed between men and women. In general, the amount of salt taken by hypertensive patients is higher than recommended allowances. Both men and women add extra amount of salt to food, women when preparing food and men at the table. Salt intake level both during preparing and eating food may be associated with occupation (unemployed), blood pressure level (systolic/diastolic ≥140/90), sex (men), age (54–59), and also patient knowledge (general).

## 1. Introduction

Hypertension and its complications are among the major health problems in many societies particularly, developing ones [1–3]. Different studies are indicative of high-salt intake among patients with hypertension [4–6]. A 30% reduction in salt intake is an international and a national goal to prevent and to control noncommunicable diseases, by the year 2020 [7, 8].

As one of the major risk factors of mortality, hypertension is considered as a leading factor for about 13% of the deaths in the world and also accounts for 12.1% of deaths in low- and moderate-income countries. In the Eastern Mediterranean region, hypertension alone accounts for 26% of deaths, while the other complications such as cardiovascular diseases and stroke, combined, lead to 31% of deaths [2, 3].

Excessive salt (sodium) intake is not only responsible for hypertension, cardiovascular diseases, and strokes but also associated directly with other diseases such as osteoporosis, nephrolithiasis, and gastric cancer [9, 10]. Therefore, control of salt intake is considered as a crucial element for a healthy and highly-qualified life [4, 11, 12]. It is believed that consuming high-fiber diets combined with reduced salt intake can play a critical role in prevention of hypertension and its fatal cardiovascular complications, as well as decreasing medical costs [13–17].

However, adding salt as a flavor to food is actually a cultural, psychological, habitual, and in turn behavioral matter [6, 18]. It happens in two phases: preparing food and eating at the table. Results of studies have illustrated multiple determinants regarding the addition of salt when preparing food or at the table [6, 19]. In most societies, people are not usually aware of the naturally occurring salt in nutrients, such as bread, milk, meat, and also other manufactured products [13, 20]. Thus, they add salt to the foods which already contain sufficient amount of salt.

In a sample of urban population in Iran, salt (sodium) intake has been reported 9.6–11.1 g/day and 2–10.3 g/day based on urinary sodium excretion and salt intake investigations, respectively [21–24]. The amount is high compared to the World Health Organization (WHO) recommendations of daily salt intake for healthy individuals (<5 g salt, equivalent to 2 gr sodium) and patients with hypertension (<4 g salt, equivalent to 1.5 gr sodium) [25, 26].

In Tehran (Iran), the blood pressure of 60% of hypertensive patients was not controlled, even though these patients were aware of their disease. However, the attitude of the patients who knew they were hypertensive was a predictive factor in salt intake [22]. Among the highly predictive of the sodium intake factors is patient knowledge of nutrients containing salt as well as their perception of illness [27]. The aims of this study were to investigate that (1) what are the predictors of salt intake among patients? (2) Do the patients diagnosed as hypertensive follow the expertise recommendations in salt taking? (3) Are there any differences between men and women in their levels of knowledge and behavior in salt taking?

## 2. Materials and Methods

### 2.1. Participants

This was a cross-sectional study conducted among a total number of 205 patients diagnosed with hypertension during the last six months, before the time of the study, and were under the coverage of 12 rural health centers of Tabriz city, Iran in Feb–May 2016. The subjects were selected based on the inclusion criteria of the aim of the study (diagnosed hypertensive and with no coexisting disease(s) and complications during the last six months, covered by the rural health centers in Tabriz and agreed to participate in the study).The patients sent written invitations to participate in the investigation and attended the health center for data collection, after the approval of number P/326/Oct. 2016. As most of the patients were illiterate, data collection was done by interviewing the patients and filling the questionnaires by interviewers.

### 2.2. Data Collection

There was a multisectional questionnaire, including anthropometric indices. Blood pressure and the amount of urine sodium and creatinine (mmol/day) were taken.

Demographic data consisted of questions regarding patient's age, sex, education level, occupation, household size, and socioeconomic status. Socioeconomic status was determined by the valid family affluence scale based on existence or nonexistence of ordinary household items, scoring from 0 to 1 and above [28]. Summing up of scores was categorized under 2 groups: low and moderate (0–14) and high (15–23).

### 2.3. Knowledge, Attitude, and Behavior

Knowledge, attitude, and behavior of patients were assessed based on the valid questionnaire [29, 30]. Four questions: (1) the patients' awareness on salt allowance and recommended for healthy people, (2) the patients' awareness on salt allowance and recommended for hypertensive people, (3) diseases induced by salt intake, and (4) the list of the common high-salt foods (23 items) were included in the knowledge questionnaire. Three questions were scored and rated based on correct and incorrect answers; 23 questions taken from a list of common high-salt foods were scored and rated based on correct (too much, much, and moderate), incorrect (too little, far too little, and do not know). To analyse the data, the replies differentiated based on “correct” and “incorrect” following a two-point scale: 1 point for “know or correct answer,” and 0 points for “don't know or incorrect answer”; the knowledge scores were computed by summing over the items and ranged from 0 to 26. The data related to patient general knowledge entered into the logistic regression model as a continuous variable.

To identify the differences between men and women, knowledge was also analyzed specifically; patient knowledge on disease related to high-salt intake and on appropriate amount of recommended salt intake for healthy and unhealthy people.

Attitude was assessed using five questions: (1) preference of food taste (salty or not salty), (2) the questions related to the healthfulness of low-salt food for healthy people, (3) the questions related to the healthfulness of low-salt food for patients, (4) in general, low-salt food is healthier, and (5) salty food is more delicious. The questions were scored based on a five point scale (strongly agree: 5, agree: 4, undecided: 3, disagree: 2, and strongly disagree: 1); the scores for attitude was computed by summing over the items for attitude which ranged from 5 to 25.

Two questions were asked to assess the patients' behavior on adding salt to food: (1) adding salt when preparing food and (2) adding salt at the table. The amount of salt added when preparing food or at the table was estimated based on home scales that was one teaspoon (1 tsp) equivalent to 4 grams. The possible answers included ≥3 tsps, 2 tsps, 1 tsp, 0.5 tsp, or not at all. For analysis of healthy and unhealthy behaviors, the scores of 0 and 1 were used, respectively.

### 2.4. Anthropometric Indices

Height (centimeter) and weight (kilogram) of the patients were measured by an adults scale to calculate body mass index (BMI), weight (kg) divided by height squared (m^2^) [31]. A trained public health technician measured systolic and diastolic blood pressure (mmHg) of all the patients who were diagnosed as hypertensive during the last six months and were taking medication, and the results were recorded and categorized according to normative (140/90), borderline, systolic ≤140, and diastolic >90, systolic >140, and diastolic ≤90, and hypertensive (equal and above 140/90). A mercury manometer was used to measure the blood pressure in sitting position.

Sodium and creatinine (mmol/day) were measured in a random urine sample on the day of data collection. The urine samples were assayed in the cooperating laboratory, by caretium were made in Hong Kong.

### 2.5. Assessment of Food Intake

A validated semiquantitative food frequency questionnaire (FFQ) was used to obtain data on consumption of high-salt food among patients [32, 33]. The FFQ consisted of 148 food items categorized in 39 food groups.

Sodium and creatinine (mmol/day) were measured in a random urine sample on the day of data collection. The urine samples were assayed in the cooperating laboratory by caretium made in Hong Kong.

### 2.6. Statistical Analyses

Data were analyzed using SPSS software 22.0 (SPSS Inc., Chicago, IL, USA). Data were summarized using frequency (percent) for categorical variables and mean and standard deviation (SD) for numerical variables. To assess differences between groups chi-squared test, Fisher's exact test, and independent samples *t*-test were used. The association between the behavior of adding salt when preparing food and also during eating food and the variables such as age, socioeconomic status, knowledge, attitude, and BMI were assessed by logistic regression modeling. Proper behavior, adding salt less than or equal to 1 tsp, was scored as 0; and the behavior of too much salt addition, more than 1tsp, was scored as 1 [34].

### 2.7. Ethical Considerations

The aims and the significance of the study were fully explained to the participants. The participants were also assured that their information would be kept confidential. Then, those willing to participate in the study signed a consent form to enter the study. Those who refused to give the urine sample, emigrants, pregnant women, having the history of psychological disorders, and suffering from metabolic diseases, renal failure, diabetes or secondary hypertension, were excluded from the study. This study was registered under the ethics code; IR.TBZMED.REC.1395.792.

## 3. Results

### 3.1. Patient's Characteristics

Out of 205 participant hypertensive patients, 62.9% were women, 91.3% illiterate or low literate, and 53.7% belonged to the low and middle socioeconomic status. There was a significant difference between men and women according to age, occupation (*P* < 0.001), BMI (*P*=0.017), and blood pressure (*P* < 0.001) (Table 1).

### 3.2. Patient's Sodium Intake and Blood Pressure

The mean (SD) of sodium intake among the patients was 4049.0 (2825.0) mg. More than half of the patients 57.1% had daily 3000 mg salt intake. Sodium intake greater than 3000 mg was observed in 58.2% of all patients and 63.4% of women with blood pressure lower than 140/90 mmHg. Among those with blood pressure ≥140/90 mmHg, 57.1% all patients had sodium intake over 3000 mg, without significant differences between men and women (*P*=0.929) (Table 2).

### 3.3. Patient's Knowledge, Attitude, and Behaviors

Distribution of frequency and percent of general knowledge and specific knowledge on disease related to high-salt intake were not significant (*P*=0.995, *P*=0.999) based on sex (Table 3). There was a significant difference between men and women according to knowledge (*P*=0.023) and behavior of adding salt during eating (*P* < 0.001) (Table 3). Patient knowledge on diseases related to high-salt intake showed significant (*P* < 0.001) based on participant blood pressure level (Table 4).

The analyses indicate that the knowledge of the participants on appropriate amount of recommended salt intake for healthy people was significant based on sex. However, the knowledge on appropriate amount of recommended salt intake for unhealthy people was significant based on hypertension levels of the patients (*P* < 0.001) (Table 5).

About half of the patients 44.9% (48.7% men and 42.6% women) added no or only 1 tsp salt when preparing food; no significant sex difference was observed (*P*=0.243). More than half of the patients 59.5% (40.8% men and 70.5% women) added 1tsp of salt at the table with a significant difference between men and women (*P* < 0.001) (Table 2).

### 3.4. Predictors of Patient Salt Intake

Results of the logistic regression test adjusted for the variables: sex, age, education level, occupation, socioeconomic status, blood pressure levels, BMI, knowledge, and attitude revealed that the adding salt beyond the dietary recommendations when preparing food (unhealthy eating behavior) had significant correlation with patient's occupation (unemployed) (OR = 4.05, 95% CI = 1.04–15.78, (*P*=0.044)), and blood pressure level (systolic/diastolic ≥140/90) (OR = 2.76, 95% CI = 1.28–5.96, (*P*=0.010)). The behavior of adding salt more than the recommended amount at the table correlated with sex (men) (OR = 4.47, 95% CI = 1.21–16.57, (*P*=0.025)), age (54–59) (OR = 0.05, 95% CI = 0.01–0.39, (*P*=0.005)), and knowledge (general) (OR = 1.06, 95% CI = 0.99–1.13, (*P*=0.05)) was also a predictor of high dietary salt intake among other predictors (Table 6).

## 4. Discussion

Hypertensive patients showed significant differences in behavioral patterns in salt taking. The differences related to sex, occupation, and blood pressure levels of the patients. About half of the patients, men and women, added optimal amount of salt (not at all or only 1 tsp salt) when preparing food, and more than half of the patients added only 1 tsp salt during eating or at the table.

The predictors that determine the amount of extra salt intake both during preparing and eating food are occupation (unemployed), uncontrolled blood pressure, sex (men), age (54–59), and also patient knowledge (general). It means unemployed patients and those with high blood pressure; systolic/diastolyic ≥140/90 add extra amount of salt while preparing food, and usually men and patient older than 54 years add extra salt at the table. In addition, the general knowledge of the patients about the customary amount of salt for hypertensive conditions, salty foods, and the association of salt with related complications play a significant role in salt intake.

Considering the specific knowledge of patients about the amount of salt intake for healthy people and related disease (hypertension), this study indicated that this amount differed significantly by sex. Yet, the patient knowledge about connection between hypertension and high-salt intake was significantly influenced by hypertensive levels of the patients rather than sex. Actually, patient knowledge on salt intake allowance for hypertensive people was not significant based on sex; instead, it differed significantly by patient blood pressure levels and occupation. It seemed hypertensive patients got knowledge about salt intake allowances because of their own health problem rather than their educational levels, socioeconomic status, and BMI. While according to the WHO studies low socioeconomic status can be a factor inducing hypertension [1], this study did not find it as an influential factor.

The results of this study came into agreement with Johnson et al. [20] in that, there was no significant correlation between knowledge and behavior of patients according to their education levels. However, coming into conflict with the results of Qin et al. [35], our findings showed that in general, hypertensive patients, no matter men and women, did not have enough knowledge about connections between hypertension and excessive salt intake. The significant specific knowledge difference related to blood pressure levels of the patients. Actually, patients became aware of the connection between too much salt intake and hypertension after becoming hypertensive. On the other hand, Sanchez et al. studied [36] knowledge and behaviors related to salt intake in urban and rural regions of three countries: Argentina, Costa Rica, and Ecuador. The results showed that the participants had a general knowledge about the amount of salt intake, that is, the risks were perceived, but the study subjects did not find themselves at risk at all, and in turn, they did not read food labels and had no tendency to learn about the salt and sodium content of processed and ready-to-eat foods [36].

Similar to the studies of Corne´lio et al. and Grime et al. [6, 19], our findings showed that there were different predictive variables about behavior of adding salt when preparing food and during eating foods. The behavior of adding salt when preparing food (more than one teaspoon) was more prevalent among women, especially unemployed women. This is because in most communities, it is usually women who prepare food. As it is evident in our study, 58% of the women were responsible for preparing food. By contrast, adding salt at the table (shaking habit) was more common among men. Another study showed that adding salt when preparing food was most prevalent among Japanese hypertensive patients. This was most likely attributed to high consumption of soy sauce and also the traditional ways of preparing food [37].

Adding salt at the table is particularly prevalent among illiterate, 54–59-years-old Iranian rural men. The percentage of men (59.2%) who added salt at the table was about twice as much as the women (25.9) and significant. Indeed, adding salt in preparing food or at the table can have habitual and psychologically unconscious reasons [6]. Women get satisfied while preparing food at the kitchen, and men get satisfied by adding salt to the food which was already salt-added [6]. The adverse behavior of adding more than one teaspoon salt when preparing food was significant based on occupation (unemployed) and blood pressure levels of patients, and during eating food or at the table based on sex (men), age (54–59), and the general knowledge of the patients.

Regarding the importance of knowledge about the recommended amounts of salt intake and its effects on behavioral patterns of healthy and hypertensive people, it is crucial to design and implement educational programs to develop healthy dietary behavior and to control blood pressure targeting both women and men hypertensive patients based on their blood pressure levels and general knowledge.

### 4.1. Limitations

Both men and women were taken as the sample of the study. The sample size was small because inclusion criteria was limited to the newly diagnosed (the last six months) hypertensive patients, and data analysis was not significant in some areas such as socioeconomic status.

## 5. Conclusion

The different behavioral patterns of salt intake was observed between men and women according to knowledge and occupation (unemployed) and age of the patients about the right amount of salt intake. In general, the amount of salt taken by men and women hypertensive patients is higher than recommended allowances. Both men and women add extra amount of salt to food, women in preparing food, and men at the table.

Salt intake level both during preparing and eating food may be associated with occupation (unemployed), blood pressure level (systolic/diastolic ≥ 140/90), sex (men), age (54–59), and also patient knowledge (general).

It is essential to provide both separate and shared educational programs for men and women to meet their different educational needs. In separate sessions, women should be educated not to add extra salt in preparing food, and men should be educated not to add salt at the table. The shared educational sessions should be held for men and women who live together to ask them to make their choice. That is, whether they want salt be added to food in preparation time or at the table. It is also beneficial to suggest them to use homemade spices instead of salt in their food. Meanwhile, the amount of salt in processed and preprepared foods should be monitored continuously to make necessary plans to prevent food factories adding excessive amount of salt to food.

## Figures and Tables

**Table 1:** Patients' characteristics.

	All	Women	Men	*P*
	*N* = 205	129 (62.9)	76 (37.1)	
Age (year)				0.001^a^
Under 33	8 (3.9)^c^	5 (9.0)^c^	3 (3.9)^c^	
34–43	19 (9.3)	18 (14.0)	1 (1.3)	
44–53	61 (29.8)	44 (34.1)	17 (22.4)	
54–59	34 (16.6)	22 (17.1)	12 (15.8)	
60 and above	83 (40.5)	40 (31)	43 (56.6)	
M (SD)^e^	56.34 (12.88)	53.2 (11.47)	61.63 (13.18)	

Education level (year)				0.190^b^
0	118 (57.6)	77 (59.7)	41 (53.9)	
1–6	69 (33.7)	41 (31.8)	28 (36.8)	
7 and above	18 (8.8)	11 (8.5)	7 (9.2)	
M (SD)^e^	2.20 (3.06)	1.98 (3.02)	2.57 (3.13)	

Occupation				<0.001^d^
Employed	77 (37.6)	7 (5.4)	70 (92.1)	
Unemployed	128 (62.4)	122 (94.6)	6 (7.9)	

FAS (item)^f^				0.41^d^
High level (15–23)	95 (46.3)	61 (64.2)	34 (35.8)	
Low/moderate level (0–14)	110 (53.7)	68 (61.8)	42 (38.2)	

BMI (kg/m^2^)^g^				0.017^b^
Underweight (18.51–25.00)	25 (12.2)^a^	10 (7.8)^a^	15 (19.7)^a^	
Normal weight (25.01–30.00)	79 (38.5)	48 (37.2)	31 (40.8)	
Overweight and obesity (30.01 and above)	101 (49.3)	71 (55.0)	30 (39.5)	

Blood pressure level (systolic/diastolic)				0.001^c^
Normotensive (under 140/90)	141 (68.8)	101 (78.3)	40 (52.6)	
Borderline^d^	43 (21)	19 (14.7)	24 (31.6)	
Hypertensive (over 140/90)	21 (10.2)	9 (7.0)	12 (15.8)	

^a, b, d^ *P* value derived from independent *t*-test, chi-squared test, Fishers' exact test, and ^c^N(%) presented. ^e^Mean (standard deviation) and ^f^Family Affluence Scale. ^a^N(%) presented and ^b,c^*P* value derived from chi-squared test and Fishers' exact test. ^d^Borderline = systolic ≤ 140 and diastolic > 90, systolic > 140 and diastolic ≤ 90.

**Table 2:** Distribution of frequency and percentage of patient blood pressure based on sex sodium intake.

	All *N* = 205			Women 129(62.9)			Men 76(37.1)		
Sodium intake^a^	<2000	2000–3000	>3000	<2000	2000–3000	>3000	<2000	2000–3000	>3000
Blood pressure^b^									
Normotensive (<140/90)	26 (18.4)	33 (23.4)	82 (58.2)	15 (14.9)	22 (21.8)	64 (63.4)	11 (27.5)	11 (27.5)	18 (45.0)
Borderline^c^	7 (16.3)	13 (30.2)	23 (5.53)	0 (0.0)	5 (26.3)	14 (7.73)	7 (29.2)	8 (33.3)	9 (5.37)
Hypertensive (≥140/90)	4 (19.0)	5 (23.8)	12 (57.1)	7 (16.3)	3 (3.33)	4 (4.44)	2 (7.16)	2 (7.16)	8 (7.66)
*P*	0.929^d^			0.226^d^			0.651^d^		

^a^mgr, ^b^systolic/diastolic, ^c^borderline = systolic ≤ 140 and diastolic > 90, systolic > 140 and diastolic ≤ 90, and ^d^*P* value derived from chi-squared test.

**Table 3:** Patients' knowledge, attitude, and behavior.

	All	Women	Men	*P*
	*N* = 205	129 (62.9)	76 (37.1)	
Knowledge (general)				0.995^a^
M(SD)^e^	13.42 (5.69)	13.43 (5.26)	13.42 (6.40)	
Knowledge on disease related to high-salt intake				0.999^b^
Correct answer ^C^	200 (97.6)	126 (97.7)	74 (97.4)	
Attitude				0.023^a^
M(SD)^e^	16.17 (3.74)	16.63 (3.38)	15.40 (4.19)	
Behavior				0.243^b^
Adding salt during cooking food				
No or less than 1 tsp^f^	92 (44.9)	55 (42.6)	37 (48.7)	
Over 1 tsp^f^	131 (55.1)	74 (57.4)	39 (51.3)	
Adding salt during eating				<0.001^b^
No or less than 1 tsp^f^	122 (59.5)	91 (70.5)	31 (40.8)	
Over 1 tsp^f^	83 (40.5)	38 (29.5)	45 (59.2)	

^a^ *P* value derived from independent *t*-test, ^b^*P* value derived from chi-squared test and Fishers' exact test, ^c^N(%) presented. ^e^Mean (standard deviation), and ^g^one teaspoon.

**Table 4:** Patient knowledge on disease related to high-salt intake based on blood pressure level.

	Blood pressure level	Borderline^b^	≥140/90^a^	All	*P* ^d^
	<140/90^a^				
Patient knowledge on disease related to high-salt intake					
Correct answer^e^	138 (97.0)	42 (97.0)	20 (95.0)	20 (98.0)	0.001

^a,c^Blood pressure = systolic/diastolic, ^b^borderline = systolic ≤ 140 and diastolic > 90, >140 and diastolic ≤ 90, and ^d^*P* value derived from chi-squared test. ^e^N(%) presented.

**Table 5:** Patient knowledge on the recommended salt intake for healthy and unhealthy people.

	Recommended salt intake for healthy people		Recommended salt intake for unhealthy people	
	Correct answer^a^ N (%)	*P* ^c^	Correct answer^b^ N (%)	*P* ^c^
Sex				
Women	11 (8.5)	<0.001^d^	43 (33.3)	0.538^e^
Men	23 (30.3)		22 (28.9)	
All	34 (16.6)		65 (31.7)	

Education level (year)		0.887^d^		0.420^d^
0	19 (16.2)		38 (31.6)	
1–6	11 (15.9)		20 (29.0)	
7 and above	4 (22.2)		7 (38.9)	

Occupation		0.002^e^		<0.001^d^
Employed	21 (61.0)		23 (35)	
Unemployed/	13 (38.0)		42 (64)	

FAS (item)^g^		0.060^d^		0.452^e^
High level (15–23)	21 (22.1)		33 (34.7)	
Low/moderate level (0–14)	13 (11.8)		32 (29.1)	

BMI (kg/m2)^**j**^		0.882^d^		0.649^e^
Underweight (18.51–25.00)	5 (20.0)		6 (24.0)	
Normal weight (25.01–30.00)	13 (15.5)		25 (31.6)	
Overweight and obesity (30.01 and above)	16 (15.8)		34 (33.7)	

Blood pressure (systolic/diastolic)		<0.001^d^		<0.001^e^
Normotensive (under 140/90)	19 (13.0)		50 (35.0)	
Borderline^k^	9 (20.0)		12 (27.9)	
Hypertensive (≥140/90)	6 (28.0)		3 (14.0)	

^a,^Patient's knowledge about recommended optimal salt intake for healthy people, ^b^patient's knowledge about recommended optimal salt intake for unhealthy people, and ^c,d,e^*P* value derived from chi-squared test and Fishers' exact test. ^g^Family Affluence Scale, ^j^body mass index, and ^k^borderline: cystolic≤140 and diastolic >90, systolic >140 and diastolic ≤90.

**Table 6:** Results of logistic regression analysis for adding salt during cooking and eating food (0 = 1 tsp^a^ or less and 1 = more than 1 tsp^a^).

	Adjusted OR 95% (CI)^b^	*P* ^c^	Adjusted OR 95% (CI)^b^	*P* ^c^
Sex				
Women	Ref.(1)^d^		Ref.(1)^d^	
Men	0.46 (0.12–1.79)	0.262	4.47 (1.21–16.57)	0.025
Age (year)				
Under 33	Ref.(1)^d^		Ref.(1)^d^	
34–43	0.29 (0.04–3.10	0.304	0.22 (0.03–1.88)	0.167
44–53	0.13 (0.02–1.21)	0.074	0.17 (0.03–1.18)	0.074
54–59	0.22 (0.02–2.49)	0.205	0.05 (0.01–.3.93)	0.005
60 and above	0.21 (0.02–1.91)	0.165	0.22 (0.03–1.54)	0.127
Education level (year)	1.04 (0.59–1.82)	0.902	0.87 (0.47–1.63)	0.673
Occupation				
Employed	Ref.(1)^d^		Ref.(1)^d^	
Unemployed	4.05 (1.04–15.78)	0.044	1.10 (0.26–3.50)	0.954
FAS (item)e				
High level (15–23)	Ref.(1)^d^		Ref.(1)^d^	
Low/moderate level (0–14)	1.10 (0.54–1.83)	0.987	1.32 (0.69–2.54)	0.398
BMI(kg/m2)^f^				
BMI (normal weight)	Ref.(1)^d^		Ref.(1)^d^	
BMI (overweight)	1.57 (0.57–4.32)	0.383	.678 (0.23–1.91)	0.479
BMI (obese)	1.51 (0.54–4.24)	0.435	1.01 (0.51–2.15)	0.897
Blood pressure (systolic/diastolic)				
Normotensive	Ref.(1)^d^		Ref.(1)^d^	
Hypertensive (≥140/90)	2.76 (1.28–5.96)	0.010	1.02 (0.41–2.11)	0.839
Knowledge (general)	1.04 (0.98–1.10)	0.227	1.06 (0.99–1.13)	0.051
Attitude	1.02 (0.94–1.12)	0.884	1.08 (0.99–1.18)	0.105

^a^One teaspoon, ^b^odds ratio 95% (confidence interval) for adding salt more than one teaspoon during food preparation, ^b^odds ratio 95% (confidence interval) for adding salt more than one teaspoon during eating (adjusted for age, sex, occupation, FAS, BMI, blood pressure levels, knowledge, and attitude), ^c^*P* value derived from logistic regression method, ^d^reference group, ^e^Family Affluence Scale, and ^f^body mass index.

## Data Availability

All raw data used for providing support the findings of this study are availble at the end of this article in the supplementary information file.

## Supplementary Materials

Supplementary Materials Additional file1: all raw data set (Data set.xls).
